# Temporal Trends in Hippocampal Sclerosis Surgery: An Observational Study From a Tertiary Epilepsy Centre

**DOI:** 10.1111/ene.70041

**Published:** 2025-01-13

**Authors:** Paola Vassallo, Vaishali Gursal, Weixi Xiong, Dong Zhou, Jane de Tisi, Roland D. Thijs, John S. Duncan, Josemir W. Sander

**Affiliations:** ^1^ Department of Clinical and Experimental Epilepsy UCL Queen Square Institute of Neurology London UK; ^2^ Stichting Epilepsie Instellingen Nederland (SEIN) Heemstede The Netherlands; ^3^ Department of Neurology Leiden University Medical Centre Leiden The Netherlands; ^4^ Chalfont Centre for Epilepsy Chalfont St Peter UK; ^5^ Department of Neurology, West China Hospital Sichuan University Chengdu Sichuan China

**Keywords:** aetiology, drug‐resistant epilepsy, outcome, temporal lobe epilepsy

## Abstract

**Objective:**

Temporal lobe epilepsy with hippocampal sclerosis (HS) is a surgically remediable syndrome. We determined temporal trends in the prevalence of hippocampal sclerosis surgeries and related factors.

**Methods:**

We analysed a prospective cohort of adults who underwent epilepsy surgery at the NHNN, London, between 1990 and 2019. HS group was compared with other pathologies. Demographics, surgical trends for HS and associations with sex, age, prior neurological insults and febrile seizures were analysed. Temporal trends were assessed by one‐way or Welch ANOVA, with post hoc analysis. Surgery latency over three decades was evaluated with the Kruskal–Wallis *H* test, using Dunn's procedure for pairwise comparisons. Chi‐squared analyses examined associations with sex, age at operation, febrile seizures, and between resection side and handedness.

**Results:**

Of 1069 people operated, 586 had hippocampal sclerosis. After increasing, surgeries declined in the last decade (from 322 to 131), as did the number of people with hippocampal sclerosis and a history of childhood febrile seizures (from 87 to 23). The median interval from epilepsy onset to surgery increased from 22 to 24 and 27 years over each decade. Female sex and febrile seizures were associated with pathology (HS vs. non‐HS) but not age at surgery, previous neurological insults, or the resection side and handedness.

**Discussion:**

Our study confirms the decline in hippocampal surgeries. This trend may be due to changes in the syndrome's natural history, possibly from improved paediatric care, and an increase in complex cases. The impact of delayed referrals, surgical risk fears and newer anti‐seizure medications remains unclear.

## 1 Introduction

Temporal lobe epilepsy (TLE) stands as the paradigm of a surgically treatable syndrome, with success rates of up to two‐thirds in selected groups [1]. Most people with TLE have mesial TLE (MTLE), and many have hippocampal sclerosis (HS), typically unilateral or bilateral but asymmetric in around a quarter of people. MRI identifies HS with atrophy and increased signal intensity on T2‐weighted images [2]. HS ‘plus’ denotes more extensive sclerosis involving the hippocampus, amygdala, parahippocampal gyrus, and the thalamus and temporal and extratemporal neocortices [3].

The hippocampus is vulnerable to epileptogenesis, and HS may follow prolonged status epilepticus; however, not all individuals with HS will necessarily develop epilepsy [4]. Understanding this condition requires a comprehensive perspective as a progressive multifactorial disease evolving under a ‘two‐hits hypothesis’ involving a pre‐existing precipitating event that can occur at any time [5].

MTLE‐HS presents a spectrum of radiological manifestations, ranging from unremarkable MRI to clear‐cut HS [6]. The natural history of MTLE is not straightforward. Some people, particularly those with onset in the first or second decade of life, may experience a benign course with effective seizure control or even remission through medical treatment, only to face a resurgence of refractory seizures necessitating surgical intervention later on [7]. The factors leading to drug resistance in this context remain to be fully elucidated.

In the case of drug‐resistant MTLE‐HS, hippocampectomy offers a 60%–70% seizure freedom rate after 2 years and 50% after 10 years [1]. Unfavourable surgical outcomes are more common if the individual has generalised seizures and evidence of dual pathology [3, 6].

Even though referral for surgical assessment is recommended for people with confirmed focal drug‐resistant epilepsy (f‐DRE) [8] and presurgical assessments increased, surgery has plateaued or decreased over the last 10–15 years [1]. Notably, there has been a decline in the proportion of individuals with MTLE‐HS [9, 10, 11, 12], and increase in individuals with normal MRI, extratemporal epilepsy, requiring intracranial recordings, and the growing use of palliative procedures like VNS [13] and minimally invasive techniques like LITT [14].

This trend has been verified in several places [9, 12, 13, 15, 16, 17, 18, 19, 20, 21, 22, 23]. The reasons for this are not fully understood. Apart from a reduction in people with incident MTLE‐HS, lack of referrals, hesitation in treating elderly individuals or changes in the natural history of the syndrome may also be contributory. A European multicentric survey of 27 centres [10] and a single‐centre Portuguese study [24] reported increased surgical procedures. This could be because some practitioners refer people earlier due to increased paediatric surgeries or how long a particular centre performs surgery. In the Portuguese cohort, this was attributed to increased tumour surgeries [24].

We analysed the 30‐year temporal trends in the prevalence of HS among people with DRE who underwent surgery at our centre. We also examined the time from the age of epilepsy onset to surgery and investigated whether sex, age at surgery, prior neurological insults and febrile seizures differed between those with and without HS. Lastly, we assessed the distribution of HS resections between the right and left hemispheres over time and explored the relationship between resection side and handedness in individuals with HS.

## 2 Methods

We analysed data from a prospectively enrolled cohort of consecutive adults with f‐DRE who underwent curative epilepsy surgery at the National Hospital for Neurology and Neurosurgery (NHNN), London, between 1 January 1990 and 31 December 2019.

### 2.1 Study Variables

We used data from the routine presurgical assessment, including video‐EEG telemetry, MRI, neuropsychological and neuropsychiatric assessment. Some individuals also had FDG‐PET, SPECT and MEG. We used data on sex, handedness, year of birth, age at epilepsy onset, history of abnormal pregnancy or delivery, childhood febrile seizures, status epilepticus in the pre‐operative period, history of convulsive seizures, prior neurological insults (head injuries or CNS infections), pre‐operative MRI diagnosis, date and type of operation and pathology of the resected brain tissue.

### 2.2 Study Outcomes

1. The numbers of HS and other neuropathological diagnoses.
2. The number of surgical procedures for HS and other diagnoses for each decade 1990–99, 2000–09 and 2010–19.
3. Latency to surgery, defined as the number of years from the age of epilepsy onset to surgery, in the HS group.
4. Differences in the HS group versus others according to sex, age at the time of surgery (< 45 years old, > 45 years old), previous neurological insults and history of febrile seizures in childhood.
5. The association between the resection side and handedness in the HS group.

The decision to operate was made by a multidisciplinary team, according to the general principles of presurgical assessment long established at NHNN, such as a well‐defined epileptogenic zone, whose resection ensures a high probability of seizure freedom with the lowest risk of complications [25]. More than 95% of the operations were performed by three consultant neurosurgeons specialising in epilepsy surgery. The specimens were examined at UCL Queen Square Institute of Neurology's Department of Neuropathology after obtaining written consent.

### 2.3 Statistical Analysis

Outcome 1: We summarised continuous variables as median values and interquartile range (IQR) and categorical data as absolute counts and percentages.

Outcome 2: People who had undergone surgery were classified into three groups on the basis of the decade in which they were operated: 1990–99, 2000–09 and 2010–19. Then, we considered two groups for each decade: HS versus non‐HS. To determine whether the number of people with HS and non‐HS operated on differed across each decade, one‐way ANOVA or Welch ANOVA was conducted. The normal distribution of data was assessed by boxplot and Shapiro–Wilk test (*p* > 0.05) for each decade subgroup and each pathology group (hippocampal sclerosis vs. other). A Levene test assessed the homogeneity of variances; in cases where it was violated, Games–Howell post hoc analysis was conducted; otherwise, a Tukey post hoc analysis was performed.

Outcome 3: The Kruskal–Wallis H test assessed differences in surgery latency among individuals with HS across the three decades. Pairwise comparisons between decades were made using Dunn's procedure (1964) with a Bonferroni correction for multiple comparisons. Statistical significance was set at *p* < 0.016.

Outcome 4: Chi‐squared analyses were conducted to test for associations between pathology and variables: sex, age at operation and febrile seizures. Associations were considered significant at *p* < 0.05.

Outcome 5: In the HS group, a chi‐squared test was conducted to test an association between handedness and side of resection. Associations were considered significant at *p* < 0.05.

Data were analysed using SPSS, version 26.0 (IBM Corp).

### 2.4 Ethics Statement

We confirm that we have read the Journal's position on issues involved in ethical publication and affirm that this report is consistent with those guidelines. Anonymised data will be shared upon reasonable request from any bona fide researcher to the corresponding author.

### 2.5 Consent

Participants consented to the investigations and surgery, and written consent was obtained for the neuropathological diagnosis. The local Research Ethics Committee has previously approved analysing acquired anonymised data without individual participant consent (UCLH Epilepsy Surgery Database, IRAS 305408).

## 3 Results

During the study period, 1669 surgeries were performed. Twenty‐five people were excluded because they had multiple surgeries; 10 pathological specimens were missing, and one pathologic diagnosis was unconfirmed.

**FIGURE 1:**
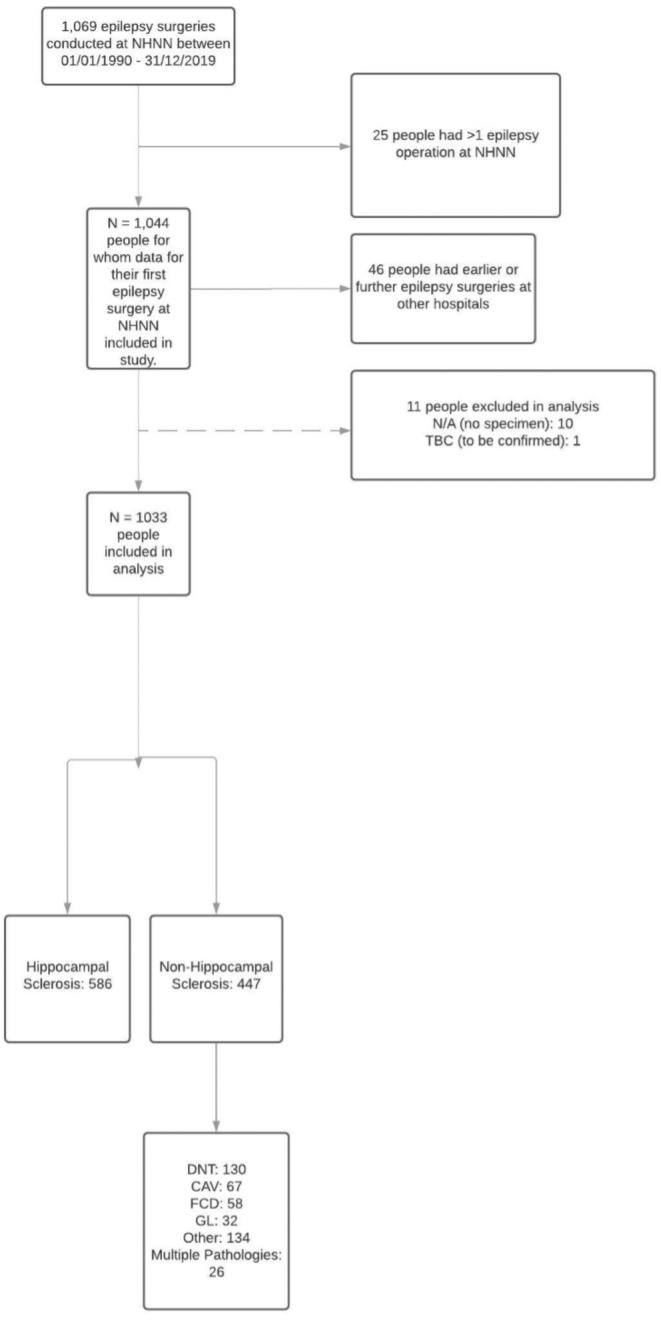
Selection of study's participants. CAV, cavernoma; DNT, dysembryoplastic neuroepithelial tumour; FCD, focal cortical dysplasia; GL, glioma; NHNN, National Hospital for Neurology and Neurosurgery.

Of the remaining 1033, 586 had HS and 447 other pathologies (Figure 1). Five hundred and fifty (53.2%) were female, the median age at epilepsy onset was 12 years, the median age at operation was 33 years, and the median latency to surgery was 20 years. Table 1 summarises demographic and clinical data. More than 70% of surgeries were performed in people < 45 years old, 324/586 (55%) of resections in the HS group involved the left hemisphere, and 484/586 (82%) of people with HS were right‐handed. Among those right‐handed, 265 underwent left resection, and 219 underwent right resection. Among left‐handed individuals, 51 had left resections, and 37 had right resections, with six left resections and five right resections in ambidextrous people. Four people were excluded from the analysis of handedness and side of resection due to lack of handedness data.

**TABLE 1:** Full demographic information is displayed for all participants used in analysis (*N* = 1033).

Total	*N* = 1033
Female sex, *n* (%)	550 (53.2)
Age of epilepsy onset, median (first quartile, third quartile), year	12 (5, 18)
Age at operation, median (first quartile, third quartile), year	33 (27, 42)
Latency to surgery, median (first quartile, third quartile)	20 (13, 29)

Prior neurological insults (PNI)^a^	Number of cases (*n* = 266)
Encephalitis	23
Head injury	175
Mild	67
Severe	97
Unclear	11
Meningitis	42
Stroke	8
Infection^b^	5
Brain malformation^c^	6
Surgical procedure^d^	1
Other^e^	2
Unconfirmed PNI	4

Childhood febrile seizures (FS)	Number of cases (*n* = 179)
< 30 min	19
≥ 30 min to < 60 min	28
≥ 60 min to < 4 h	35
≥ 4 h	14
Unknown duration	83

^a^ Categorisation of PNI and the number of people who have identified or unconfirmed PNIs by a neurologist prior to surgery.

^b^ ‘Infection’ includes people with septicaemia, mastoiditis, *Escherichia coli* , cranial pressure‐pilocystic AS‐infection and mumps.

^c^ ‘Brain malformation’ includes people with identified Dandy–Walker syndrome, left temporal lobe cyst, lesion, neuroectodermal tumour, congenital hydrocephalus and LF angioma.

^d^ ‘Surgical procedure’ involves a person who had a right temporal arteriovenous malformation removal.

^e^ ‘Other’ includes people diagnosed with Henoch–Schönlein Purpura and meningoencephalitis.

### 3.1 Temporal Trends of HS and Factors Associated With Pathology

**FIGURE 2:**
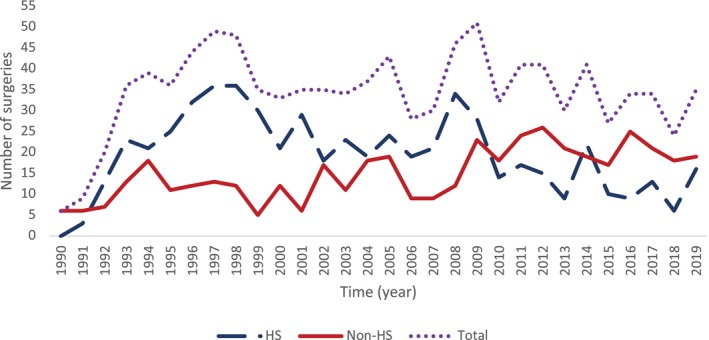
Number of HS and non‐HS over the time. The number of HS surgeries performed increases in the first two decades and then decreases in the last decade. Non‐HS surgeries performed increase over all three decades and exceed the number of HS surgeries in 2010. HS: *N* = 586, non‐HS: *N* = 447. Decade 1 (HS: *N* = 219, 68%; non‐HS: *N* = 103, 32%). Decade 2 (HS: *N* = 236, 63.4%; non‐HS: *N* = 136, 36.6%). Decade 3 (HS: *N* = 131, 38.6%, non‐HS: *N* = 208, 61.4%).

**FIGURE 3:**
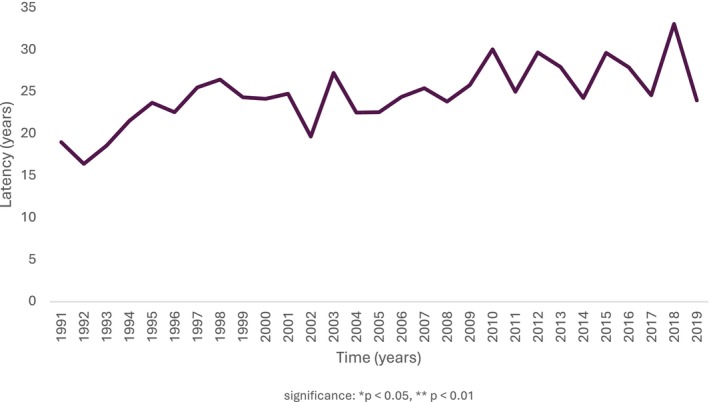
Latency from epilepsy onset to surgery in people with HS. Over time, the latency to surgery increased (Decade 1: *N* = 219, Decade 2: *N* = 236, Decade 3: *N* = 131; Group 1 vs. Group 3 **p = 0.012). Levels of significance *p < 0.05, **p < 0.01.

Overall surgical activity remained stable over time; however, HS surgeries decreased over time, with a significantly increased delay from epilepsy onset to surgery (Table 2, Figure 2). The median latency from epilepsy onset to surgery in the population was 20 years (IQR 13, 29). The number of non‐HS surgeries increased significantly from Decade 1 to Decade 3 (Table 2, Figure 3). A comparison between groups categorised by pathology into HS and non‐HS (including dysembrioplastic neuroepithelial tumours, cavernomas, focal cortical dysplasia and gliomas) suggests that sex and history of childhood febrile seizures were significant factors associated with pathology (Table 3). More females underwent HS, and more males underwent non‐HS surgery. The majority of people in both groups had no history of febrile seizures, but chi‐squared analyses showed a strong association between HS and febrile seizures (*p* < 0.001). The proportion of febrile seizures in the HS group decreased across decades, with a notable decrease in the third decade, though this trend did not reach statistical significance (Table 2). Besides the history of febrile seizures, the majority of people (75%) had no prior neurological insults (Table 1).

**TABLE 2:** Temporal trends in hippocampal sclerosis (HS) surgery and other pathologies, with pairwise comparisons across decades using Tukey's post hoc analysis and corresponding *p* values.

	HS (*N*, %)	Other pathologies (*N*, %)	Latency to HS surgery (median, years)	Total surgeries (*N*, %)	History of prolonged early childhood convulsions
					HS *n* = 163 vs. other pathologies *n* = 16, **p < 0.01
Decade 1: 1990–99	219 (68%)	103 (32%)	22.55	322 (100)	87
Decade 2: 2000–09	236 (63.4%)	136 (36.6%)	24.23	372 (100)	69
Decade 3: 2010–19	131 (38.6%)	208 (61.4%)	27.92	339 (100)	23
*Pairwise comparisons*					
Decade 1 vs. Decade 2	*p* = 0.921	*p* = 0.223	*p* = 0.457		
Decade 2 vs. Decade 3	*p* = 0.001**	*p* = 0.003*	*p* = 0.055		
Decade 1 vs. Decade 3	*p* = 0.15	*p* = 0.001**	*p* = 0.012*		

*Note:* The latency from epilepsy onset to HS surgery was assessed, with pairwise comparisons across decades conducted using Dunn's post hoc analysis and corresponding *p* values. Temporal trends of history of prolonged early childhood convulsions, with chi‐squared test and *p* values indicating the level of significance (*p < 0.05, **p < 0.01).

**TABLE 3:** Factors associated with pathology (HS vs. other pathology groups).

	HS	Non‐HS	Test used
Sex (male vs. female)	244 vs. 342	239 vs. 208	Chi‐squared *p* = 0.001*
Prior neurological insult (PNI) (yes vs. no)	144 vs. 442	110 vs. 337	Chi‐squared *p* = 0.990
Febrile seizures (yes vs. no)	163 vs. 423	16 vs. 431	Chi‐squared *p* < 0.001**
Age at operation			
< 45 vs. > 45 years	465 vs. 121	370 vs. 77	Chi‐squared *p* = 0.166
*Handedness and side of operation*			
Right‐handed: left vs. right resection	265 vs. 219		Chi‐squared *p* = 0.856
Left‐handed: left vs. right resection	51 vs. 37		
Ambidextrous: left vs. right resection	6 vs. 5		

*Note:* Handedness and side of resection are reported only for the HS group. Comparison were conducted using chi‐squared tests with *p* values indicating the levels of significance (*p < 0.05, **p < 0.01).

## 4 Discussion

Overall surgical activity was stable over time, with a non‐significant increase in HS surgeries until 2009, followed by a significant decrease in the subsequent decade (Figure 2). Our results reflect an international trend, with an overall decline in resective neurosurgery for epilepsy in the UK [13, 26], Europe [9, 10, 11, 24, 27, 28, 29, 30, 31], United States [16, 17, 18, 19, 21, 22, 32], Canada [15] and Australia [32].

The advent of MRI technology and improved protocols for diagnosing and classifying HS [12, 31, 33] and the referral of a backlog of individuals with HS could explain the initial increase in HS surgeries. The reason for the subsequent decline in HS surgery is not straightforward. This is hypothesised as a lack of eligible candidates among people with f‐DRE and the backlog of ‘good candidates’ being treated.

Improvements in managing prolonged seizures [34, 35, 36, 37] and immunisation programmes in the paediatric population may have reduced the incidence of HS by preventing infection‐related fevers and associated febrile seizures [34]. We found a decline in the number of people with HS and a history of prolonged early childhood seizures across each successive decade. At the same time, prior neurological insults, which occurred in a minority of our population, were not associated with HS.

Understanding the impact of disease‐modifying factors or their combinations on the decreasing incidence of HS requires population‐based epidemiological studies encompassing surgical and non‐surgical candidates with HS in low‐ and high‐income countries.

The burden of comorbidities in older people and a reluctance to have surgery [27, 31], along with the onset of refractory epilepsy in the early decades of life, may explain why many surgeries in our cohort occurred in younger individuals. Retrospective studies in adults show inconsistent trends in age distribution, with some reporting more procedures in younger people and others a higher frequency of surgery in older populations [15, 28]. Conversely, as most f‐DRE starts in childhood, surgery in paediatric populations is increasing [20, 38, 39, 40], but its impact on adult surgery rates remains uncertain.

The median latency to surgery for HS increased by 4 years between the first and the third decade (Table 2, Figure 3). Although some studies align with our findings, ranging between 2 [31] and 5 [28] years, others diverge, showing a declining trend between 2.6 [9] and 5.2 [10] years. The reasons for these contrasting results are unclear, but variations in referral practices to epilepsy centres could be responsible [41]. This delay could also be due to the prolonged periods of remission, a longer work‐up for more complex cases and the availability of newer anti‐seizure medications, with neurosurgery being regarded as a last resort [42], with up to a quarter of those with HS achieving seizure freedom [43, 44]. The slight improvement in the overall seizure freedom rate over time [10, 11, 27] suggests that the advent of newer anti‐seizure medications (ASM) may influence the decrease in HS surgeries. Comparative studies of referral latency of people with surgically treatable syndromes (e.g., dysembryoplastic neuroepithelial tumour and focal cortical dysplasia) could contribute significantly to understanding the impact of newer ASMs.

It is also possible that the MTLE‐HS and MTLE‐HS ‘plus’ cases are now perceived as less challenging neurosurgically and are more likely to be managed in regional neurosurgery centres with fewer referrals to supraregional centres [9, 10, 11, 31]. Comprehensive, national data are needed to clarify this issue. A UK national survey of epilepsy surgery activity in 2011 showed decreased temporal lobe resections and an increase in VNS implants [13]. Furthermore, such studies are needed.

A strength of our study is the prospective data from a comprehensive surgical database that systematically collects pre‐ and post‐surgical data. Standardised protocols ensure methodological consistency in assessing surgery candidates and diagnostic certainty based on histopathological criteria.

Limitations include the focus on operated adults, mainly from the UK, which affects the generalisability to paediatric populations and other countries. Our analysis did not include data on people with HS who were not assessed for surgery. Additionally, we did not consider data on referral patterns and social and ethnic background.

## 5 Conclusion

Centres in high‐income countries have reported declining numbers of TLE with HS surgeries. Our results align with this. Reasons are likely to be multifactorial, including treatment of the backlog of adults with HS, a reduced HS incidence and more childhood epilepsy surgery. In parallel, there is an increase in people referred with complex refractory focal epilepsies and a range of causes and comorbidities. This underscores the need to explore other possible disease‐modifying factors, akin to how febrile seizures were discovered to be linked with hippocampal sclerosis in the past, and perhaps incorporate routine genetic assessments for f‐DRE. Factors, such as delayed referrals and the influence of newer anti‐seizure medications, may also play a role in this decline. Future research should focus on comparative studies of other surgically treatable syndromes in high, middle and low‐income countries.

## Author Contributions

**Paola Vassallo:** conceptualization, writing – original draft, writing – review and editing. **Vaishali Gursal:** conceptualization, data curation, formal analysis, writing – review and editing. **Weixi Xiong:** conceptualization, supervision, writing – review and editing. **Dong Zhou:** review and editing. **Jane de Tisi:** data curation, review and editing. **Roland D. Thijs:** review and editing. **John S. Duncan:** conceptualization, review and editing. **Josemir W. Sander:** conceptualization, supervision, formal analysis, writing – review and editing.

## Conflicts of Interest

RDT has received personal compensation for serving on the Advisory Boards or Speaker's Bureau for Xenon, Theravance, Novartis, Esai, Angelini Pharma and UCB Pharma. JWS or his department has received grants from Esai, Angelini Pharma and UCB. He has received personal compensation for serving on the Advisory Boards or Speaker's Bureau for UCB and Angelini Pharma. All other authors declare no conflicts of interests.

## Data Availability

The data that support the findings of this study are available from the corresponding author upon reasonable request.
